# An Unsupervised Situation Awareness Framework for UAV Sensor Data Fusion Enabled by a Stabilized Deep Variational Autoencoder

**DOI:** 10.3390/s26010111

**Published:** 2025-12-24

**Authors:** Anxin Guo, Zhenxing Zhang, Rennong Yang, Ying Zhang, Liping Hu, Leyan Li

**Affiliations:** School of Air Traffic Control and Navigation, Air Force Engineering University, Xi’an 710051, China; anxing0519@163.com (A.G.);

**Keywords:** sensor fusion, situation awareness, variational autoencoder, unsupervised learning, mixture density network, time-series data processing

## Abstract

Effective situation awareness relies on the robust processing of high-dimensional data streams generated by onboard sensors. However, the application of deep generative models to extract features from complex UAV sensor data (e.g., GPS, IMU, and radar feeds) faces two fundamental challenges: critical training instability and the difficulty of representing multi-modal distributions inherent in dynamic flight maneuvers. To address this, this paper proposes a novel unsupervised sensor data processing framework to overcome these issues. Our core innovation is a deep generative model, VAE-WRBM-MDN, specifically engineered for stable feature extraction from non-linear time-series sensor data. We demonstrate that while standard Variational Autoencoders (VAEs) often struggle to converge on this task, our introduction of Weighted-uncertainty Restricted Boltzmann Machines (WRBM) for layer-wise pre-training ensures stable learning. Furthermore, the integration of a Mixture Density Network (MDN) enables the decoder to accurately reconstruct the complex, multi-modal conditional distributions of sensor readings. Comparative experiments validate our approach, achieving 95.69% classification accuracy in identifying situational patterns. The results confirm that our framework provides robust enabling technology for real-time intelligent sensing and raw data interpretation in autonomous systems.

## 1. Introduction

**Figure 1:**
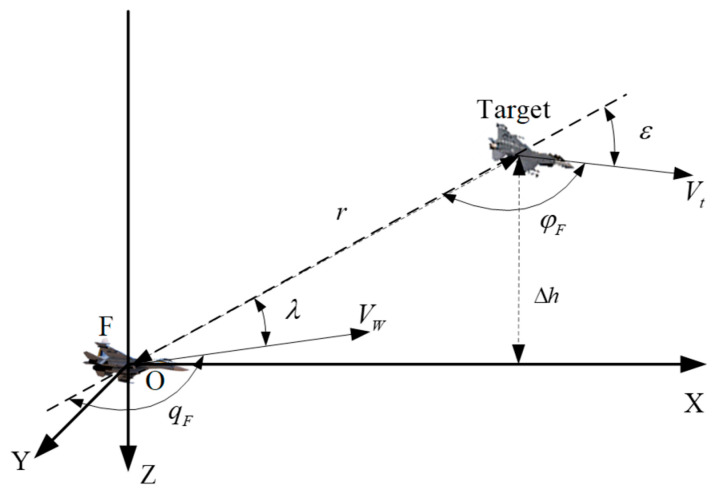
Position relationship between both agents in Relative state estimation.

In recent years, Unmanned Aerial Vehicles (UAVs) have been increasingly deployed in complex and dynamic environments [1]. The success of these missions hinges on the UAV’s ability to autonomously perceive and comprehend its operational environment—a capability known as situation awareness (SA) [2]. In a typical engagement, such as the 1V1 scenario depicted in Figure 1, a modern UAV is equipped with a plethora of sophisticated sensors, including Inertial Navigation Systems (INS), radar, and electro-optical pods. These sensors generate exponential volumes of high-dimensional, non-linear time-series data [3]. Consequently, the challenge has shifted from data acquisition to intelligent sensor data interpretation and fusion. The development of robust systems capable of fusing and understanding these multi-variate sensor streams in real-time has become a paramount challenge [4,5].

Traditionally, SA relied on methods like fuzzy Bayesian networks [6,7], which struggle with the high-dimensional, non-linear, and temporal nature of modern UAV data. To address this, the field has shifted towards deep learning. Recent advancements include the use of Transformer-based architectures to model long-range temporal dependencies in flight trajectories [8,9]. Other promising directions involve Generative Adversarial Networks (GANs) for detecting anomalous flight patterns [10] and contrastive learning frameworks to learn discriminative representations from unlabeled data in a self-supervised manner [11,12,13]. These approaches have demonstrated significant progress in automatically extracting meaningful features from complex sensor streams.

However, despite their success, these state-of-the-art methods often overlook critical practical challenges when applying deep generative models, a cornerstone of unsupervised representation learning. For instance, the popular Variational Autoencoder (VAE) [14,15,16,17], while powerful in theory, is notoriously difficult to train on complex, real-world time-series data. They suffer from critical training instability and are prone to converging to poor local minima, a limitation we quantitatively analyze in Section 6.3, where standard VAEs exhibited non-convergence during our experiments. Furthermore, both discriminative and generative models often make a simplifying unimodal assumption, which is inadequate for UAV engagement scenarios where a single tactical state can manifest through multiple, distinct sensor patterns (i.e., a multi-modal distribution)

To address these specific gaps, this paper proposes an improved deep generative framework, termed VAE-WRBM-MDN, built upon a Bidirectional Long Short-Term Memory (BLSTM) backbone to effectively model temporal dynamics. Our architecture is engineered to be a robust, specialized solution through three key contributions. First, to solve the critical problem of training instability, we introduce Weighted-uncertainty Restricted Boltzmann Machines (WRBM) [18,19,20,21,22] for layer-wise pre-training. This ensures stable convergence by guiding the model to a superior region of the parameter space, a step we found essential for our task. Second, to capture the complex, multi-modal nature of UAV data, we integrate a Mixture Density Network (MDN) [23,24] into the VAE decoder, allowing it to model complex conditional output distributions far more accurately than standard approaches. Third, our entire framework operates in a fully unsupervised manner, which is crucial given the infeasibility of acquiring large, comprehensively labeled datasets for all possible aerial situations [25,26,27]. This synergistic design transforms the VAE from a general tool into a robust solution specifically tailored for the challenges of UAV situation awareness.

Building on this integrated design, the main contributions of this paper are summarized as follows:

1. We propose a novel, fully unsupervised situation awareness framework built on a BLSTM backbone to effectively capture temporal dynamics from high-dimensional UAV sensor data.
2. We introduce a WRBM-based pre-training strategy that fundamentally solves the training instability of deep VAEs on this complex task, transforming it from an unusable baseline into a robust feature extractor.
3. We enhance the model’s representational power by integrating an MDN into the decoder, enabling it to accurately model the multi-modal distributions inherent in real-world engagement data.
4. We develop a hybrid classification process that synergizes data-driven clustering with a knowledge-based refinement step, ensuring the final situational assessment is both mathematically robust and operationally sound.

The remainder of this paper is organized as follows: In Section 1, factors of situation awareness are analyzed according to the characteristics of relative motion dynamics, and the model of situation awareness is built. In Section 2, a new model, deep VAE-WRBM-MDN, is proposed to improve the performance of feature extraction. In Section 3, two clustering algorithms: k-means++ and density peak are applied to cluster extracted features into four classes: our superiority $V_{1}$, the Target’s superiority $V_{2}$, neutrality $V_{3}$ and balance of forces $V_{4}$. In Section 4, combining Heuristic Evaluation Function with experts’ interpretation, the classification results are amended. In the experiments, feature extraction algorithms, principal components analysis (PCA), VAE, VAE-WRBM and VAE-WRBM-MDN are matched with classification algorithms, k-means++ and density peak respectively, forming 10 horizontal and vertical contrast experiments. Indicators like classification accuracy, the run time of single-group data and the occupancy peak of CPU are selected to evaluate the effectiveness of the algorithm.

## 2. Model Construction of Relative State Estimation Situation

### 2.1. Modeling of Multi-Variate Sensor Data for Relative States

In a 1V1 engagement scenario, the state of our UAV can be represented by a vector $S_{own}\;={\left[p_{x}\;,p_{y}\;,p_{z}\;,v,\psi ,\theta ,\phi \right]}^{T}$, where $\;\left(p_{x},p_{y},p_{z}\right)$ are its position coordinates,  v is its velocity, and $\;\left(\psi ,\theta ,\phi \right)$ are its Euler angles. The state vector is derived from the fusion of onboard GPS, IMU, and radar tracking data. The raw sensor measurements are processed to yield relative parameters, as summarized in Table 1.

The primary task of the UAV’s situation awareness system is to determine its tactical advantage relative to a Target (which could be another UAV or a ground object) based on parameters like position, attitude, and motion state. The geometric relationship between the UAV and the Target is shown in Figure 1. In the UAV axis OXYZ, the center of gravity of the UAV is taken as the origin O, X-axis is in accordance with the longitudinal axis of the UAV, pointing to the head direction, Y-axis perpendiculars to the symmetry of the UAV, pointing to the right side; Z-axis lies in the symmetry of the UAV and perpendicular to the longitudinal axis, pointing downwards. Azimuth angle λ refers to the angle between Target’s line of sight and speed direction of our UAV, Target entry angle ε refers to the angle between extension line of Target’s line of sight and speed direction of Target UAV, η refers to the angle between velocity vectors of two UAVs.

### 2.2. Classification of Situation Space

**Figure 2:**
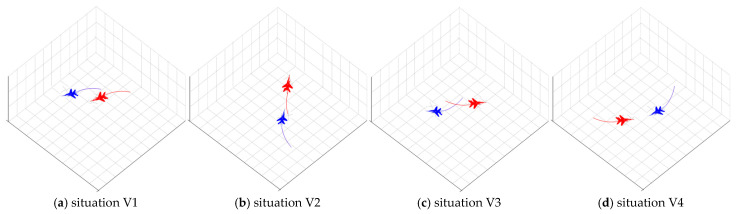
Four kinds of classic aerial encounter situations.

Based on established tactical doctrine, the engagement situation can be qualitatively categorized into four classic archetypes, as shown in Figure 2. It is crucial to note that these are not predefined classes with rigid, quantitative boundaries. Instead, they represent tactically meaningful concepts that we expect our unsupervised framework to discover as distinct clusters from high-dimensional data.

➢ $V_{1}$ (Advantageous): Our UAV is in an offensive position (e.g., at the Target’s rear), with superior speed or altitude, meeting the conditions for a successful engagement.
➢ $V_{2}$ (Disadvantageous): The Target is in an offensive position relative to our UAV, posing a significant threat.
➢ $V_{3}$ (Neutral-Passive): The distance between the UAV and the Target is large, and neither side is in a position to engage effectively. This is often the initial approach phase.
➢ $V_{4}$ (Mutual Engagement/Balance of Power): Both the UAV and the Target are in a position to engage each other, representing a high-stakes, balanced confrontation.

This section has defined the high-dimensional feature space that describes physical engagement. The primary challenge, which the following sections address, is to develop a deep learning methodology capable of automatically extracting a low-dimensional, meaningful representation from this complex data stream without relying on manual labels.

## 3. Feature Extraction

The Time-series sensor data sequence is represented by a time series $X=\{x^{\left(1\right)},x^{\left(2\right)},\ldots ,x^{\left(L\right)}\}$ of length L, where $x^{\left(i\right)}\in R^{200}$. In order to avoid dimensionality curse and improve the accuracy of classification, a new unsupervised deep learning model—VAE-WRBM-MDN is proposed to extract key features from situation data.

### 3.1. Data Processing

Raw Time-series sensor data is often corrupted by anomalies (outliers) and noise, which can be attributed to volatile operational environments and sensor instability. Therefore, data cleaning is a necessary preprocessing step before classification. Our data cleaning process consists of the following steps:

(1) Anomaly Elimination

We employ the Isolation Forest algorithm, a non-parametric and unsupervised method, for anomaly detection. This algorithm is particularly well-suited for rapidly processing large datasets and has been shown to outperform ORCA, LOF, and Random Forest in similar tasks. In this study, we use it to identify and remove outliers from the Time-series sensor data.

(2) Coordinate conversion

In order to achieve quantitative analysis of the relative situation among UAVs, the WGS-84 geodetic coordinate system obtained by GPS is transformed into China’s national coordinate system.

(3) Normalization

As for data of large range, there will be problems of slow convergence speed and long training time emerging in deep learning algorithms. So, the data is normalized to maintain the uniformity of the data range:

(1) $$x^{*}=\frac{x-x_{\min}}{x_{\max}-x_{\min}}$$

### 3.2. VAE Model with Bidirectional Long Short-Term Memory (BLSTM)

With a typical variational inference framework, VAE can be used to learn low dimension latent variables z, given the visible variables x. In contrast to standard autoencoder, x and z in VAE are stochastic variables, the output of VAE are parameter distribution values, and the weights of VAE represent the variational parameters ϕ and θ.

It is possible for VAE to get the encoding distribution $q_{\phi }(z\left|x\right.)$ given the visible variables, a prior $p\left(z\right)$ over the latent variables, and the decoding distribution $p_{\theta }(x\left|z\right.)$ given the latent variables. Where $q_{\phi }(z\left|x\right.)$ estimates the true and unknown posterior $p(z\left|x\right.)$, and $p_{\theta }(x\left|z\right.)$ implements the generative model.

BLSTM is suitable to work with time series for its superiority in obtaining information forward and backward simultaneously. Compared with LSTM, BLSTM reveals higher precision and convergence speed in time-series prediction. Thus, it is applied to be the basic model of encoder and decoder. Meanwhile, in order to improve learning ability, multiple BLSTMs are stacked to construct deep BLSTM network. As for BLSTM with single layer, forward sequence, backward sequence and the output are:

(2) $$\begin{array}{l} {\vec{h}}_{t}=g(W_{x\vec{h}}x_{t}+W_{\vec{h}\vec{h}}{\vec{h}}_{t-1}+b_{\vec{h}})\\ {\overset{\leftarrow }{h}}_{t}=g(W_{x\overset{\leftarrow }{h}}x_{t}+W_{\overset{\leftarrow }{h}\overset{\leftarrow }{h}}{\overset{\leftarrow }{h}}_{t+1}+b_{\overset{\leftarrow }{h}})\\ y_{t}=g(W_{\vec{h}y}{\vec{h}}_{t}+W_{\overset{\leftarrow }{h}y}{\overset{\leftarrow }{h}}_{t}+b_{y}) \end{array}$$

where g(⋅) represents the activation function, $W_{mn}$ represents the weight, and $b_{n}$ represents bias. 

For visual variables and its corresponding latent variables, we assume the joint probability distribution factorizes over time as follows, where the generative and inference models are parameterized by deep BLSTM networks:

(3) $$\begin{array}{l} p(x_{1:T},z_{1:T})=\\ \prod_{t=1}^{T}p_{\theta }(x_{t}\left|({\vec{h}}_{t}^{g}\right.(x_{t-1},z_{t},{\vec{h}}_{t-1}^{g}),{\overset{\leftarrow }{h}}_{t}^{g}(x_{t-1},z_{t},{\overset{\leftarrow }{h}}_{t+1}^{g}))\\ \;\cdot p_{\phi }(z_{t}\left|({\vec{h}}_{t}^{p}\right.(z_{t-1},{\vec{h}}_{t-1}^{p}),{\overset{\leftarrow }{h}}_{t}^{p}(z_{t-1},{\overset{\leftarrow }{h}}_{t+1}^{p})) \end{array}$$

This equation is crucial as it mathematically formalizes the temporal generative process our model assumes for the time-series sensor data. It explicitly defines how the joint probability distribution factorizes over time, showing how the current observation is generated from the current latent variable and historical context (via the BLSTM’s hidden states). The decoding model, $q_{\phi }(z\left|x\right.)$, the generative model $p_{\theta }(x\left|z\right.)$, and the priors are all implemented by multiple BLSTMs with weights θ and ϕ, and hidden states ${\vec{h}}_{t}^{g}$, ${\overset{\leftarrow }{h}}_{t}^{g}$, ${\vec{h}}_{t}^{p}$ and ${\overset{\leftarrow }{h}}_{t}^{p}$. The output and input data of these BLSTMs are corresponding to all the parameters of the distribution, such as input data $z\left|x\right.\sim N(\mu_{x},\sigma_{x}^{2})$, then these BLSTMs output μ and σ.

The objective of VAE with BLSTMs is to maximize the following lower variational bound of the log likelihood $L_{\left(VAE\right)}^{v}$ with respect to the parameters θ and ϕ:

(4) $$\begin{array}{l} \log p_{\theta }(x)\geq L_{\left(VAE\right)}^{v}=E_{q_{\phi }(z\left|x\right.)}[\log\frac{p_{\theta }(x\left|z\right.)}{q_{\phi }(z\left|x\right.)}]\\ =E_{q_{\phi }(z\left|x\right.)}[\log p_{\theta }(x\left|z\right.)]-KL(q_{\phi }(z\left|x\right.)\left\|p(z)\right.)\\ \approx \frac{1}{L}\sum_{i=1}^{L}\log(p(x^{i}|z^{\left(i,l\right)}))-KL(q_{\phi }(z\left|x\right.)\left\|p(z)\right.) \end{array}$$

This objective function, known as the Evidence Lower Bound (ELBO), is central to the training of any VAE. It consists of two key terms. The first, $E_{q_{\phi }(z\left|x\right.)}[\log p_{\theta }(x\left|z\right.)]$, is the reconstruction loss, measuring how accurately the decoder can regenerate the input data x from its latent representation z. The second, $-KL(q_{\phi }(z\left|x\right.)\left\|p(z)\right.)$, is the Kullback-Leibler (KL) divergence, which acts as a regularization term. It penalizes the model if the learned posterior distribution $q_{\phi }(z\left|x\right.)$ deviates too far from a predefined prior p(z) (typically a standard normal distribution), thus encouraging a smooth and well-organized latent space. In the context of our work, this equation represents the standard VAE objective that serves as a baseline, which we aim to stabilize and enhance. To optimize this objective function via gradient descent, we employ the reparameterization trick, which allows the gradient to be backpropagated through the stochastic sampling process by re-expressing the latent variable z as a deterministic function of the encoder’s output and a random noise variable. In the context of our work, this equation represents the standard VAE objective that serves as a baseline, which we aim to stabilize and enhance.

### 3.3. Optimizing VAE Initialization with WRBM Pre-Training

As identified in the Introduction and confirmed by our preliminary experiments where a standard VAE failed to converge, the training instability of deep VAEs is a primary obstacle to their application on complex time-series data. This instability often stems from poor weight initialization, which can lead to vanishing/exploding gradients or convergence to suboptimal local minima. To address this fundamental problem, we introduce a greedy, layer-wise pre-training strategy using Weighted-uncertainty Restricted Boltzmann Machines (WRBM) to find a highly effective set of initial weights for the entire VAE network.

The pre-training process unfolds in a sequential manner, treating the deep VAE as a stack of individual layers to be initialized one by one. The procedure is as follows:

1. Encoder Pre-training (Bottom-up):

Layer 1: A WRBM is constructed where its visible layer matches the dimension of the input data and its hidden layer matches the dimension of the VAE encoder’s first hidden layer. This WRBM is trained on the raw input data until it learns to effectively reconstruct the input. The learned weight matrix from this WRBM is then used to initialize the weights of the first layer of the VAE encoder.

Layer 2 to N: Once the first WRBM is trained, the training data is passed through it to obtain the activations of its hidden units. These activations then serve as the input data for training a second WRBM, which corresponds to the second layer of the VAE encoder. This process is repeated greedily for all subsequent layers of the encoder, with the output of one trained layer becoming the input for the next.

2. Decoder Pre-training (Top-down): The same layer-wise strategy is applied to the decoder, but in reverse. The final hidden representation from the encoder pre-training is used as the initial input to train the WRBM corresponding to the top-most layer of the decoder, and the process continues downwards until the output layer is reached.

Clarification on Stochasticity: It is crucial to distinguish between the roles of stochasticity in WRBM and VAE. The WRBM, as a probabilistic energy-based model, is used here for a deterministic purpose: to find a single, high-quality set of initial weights. Once the pre-training is complete, these learned weights are fixed as the starting point for the VAE. The stochasticity inherent to the VAE’s operation—namely, the sampling of the latent variable z from the posterior distribution q(z|x) via the reparameterization trick—proceeds from this well-initialized state. Therefore, the WRBM does not introduce additional randomness into the VAE’s final inference process; rather, it ensures that the VAE’s gradient-based optimization begins in a favorable region of the parameter space, dramatically improving the likelihood of stable and meaningful convergence.

By following this procedure, the entire deep VAE is initialized with weights that have already been learned to extract hierarchical features from the data. This provides the subsequent end-to-end fine-tuning process with a powerful head start, effectively circumventing the convergence issues that plague randomly initialized deep VAEs.

### 3.4. MDN-VAE Model

To overcome the limited representational power of a standard VAE decoder, which assumes a simple unimodal output distribution, we introduce a Mixture Density Network (MDN). The specific advantage of MDN is its ability to model complex, multi-modal data distributions, which is essential for accurately reconstructing UAV sensor data. MDN can obtain the probability distribution of each output value through weighted sum of multiple probability distribution functions at the output layer. However, compared with MDN, VAE ignores the weights of different outputs and directly outputs distribution parameters. Therefore, MDN is introduced into VAE model.

For the output layer, the Gaussian function is chosen as probability density function (PDF), which can be defined as:

(5) $$P(y_{t}|N_{t})=\sum_{c=1}^{C}\omega_{t}^{c}N\left(y_{t}|\mu_{t}^{c},\sigma_{t}^{c},\rho_{t}^{c}\right)$$

where $y_{t}$ represents the ground truth, C represents the number of PDF, $\omega_{t}^{c}$ represents the weight of the PDF, N(⋅) represents the Gaussian function.

The parameters in N(⋅) are normalized as follows:

(6) $$\left\{\begin{array}{l} \mu_{t}^{c}={\tilde{\mu }}_{t}^{c}\\ \omega_{t}^{c}=\frac{\exp\left({\tilde{\omega }}_{t}^{c}\right)}{\sum_{i=1}^{C}\exp\left({\tilde{\omega }}_{i}^{c}\right)}\\ \sigma_{t}^{c}=\exp\left({\tilde{\sigma }}_{t}^{c}\right)\\ \rho_{t}^{c}=\tanh\left({\tilde{\rho }}_{t}^{c}\right) \end{array}\right.$$

where ${\tilde{\mu }}_{t}^{c}$, ${\tilde{\sigma }}_{t}^{c}$, ${\tilde{\omega }}_{t}^{c}$ and ${\tilde{\rho }}_{t}^{c}$ are mean value of output, variance of output, weight of PDF and the correlation of the Gaussian component respectively. 

The objective of MDN-VAE is to maximize the following lower variational bound of the log likelihood $L_{\left(MDN-VAE\right)}^{v}$:

(7) $$\begin{array}{l} L_{\left(MDN-VAE\right)}^{v}=E_{q_{\phi }(z\left|x\right.)}[\log p_{\theta }(x\left|z\right.)]-KL(q_{\phi }(z\left|x\right.)\left\|p(z)\right.)\\ \approx \frac{1}{L}\sum_{i=1}^{L}\log(\omega^{i}p(x^{i}|z^{\left(i,l\right)}))-KL(q_{\phi }(z\left|x\right.)\left\|p(z)\right.) \end{array}$$

The key difference from the standard VAE objective in Equation (4) is that the reconstruction term $\log(\omega^{i}p(x^{i}|z^{\left(i,l\right)}))$ is now computed using the probability density of the Gaussian mixture output by the MDN. This modification is the mathematical embodiment of our model’s enhanced capability. It allows the objective function to reward the model for accurately capturing multi-modal data distributions, directly addressing a core limitation of conventional VAEs that assume a simple, unimodal output.

**Figure 3:**
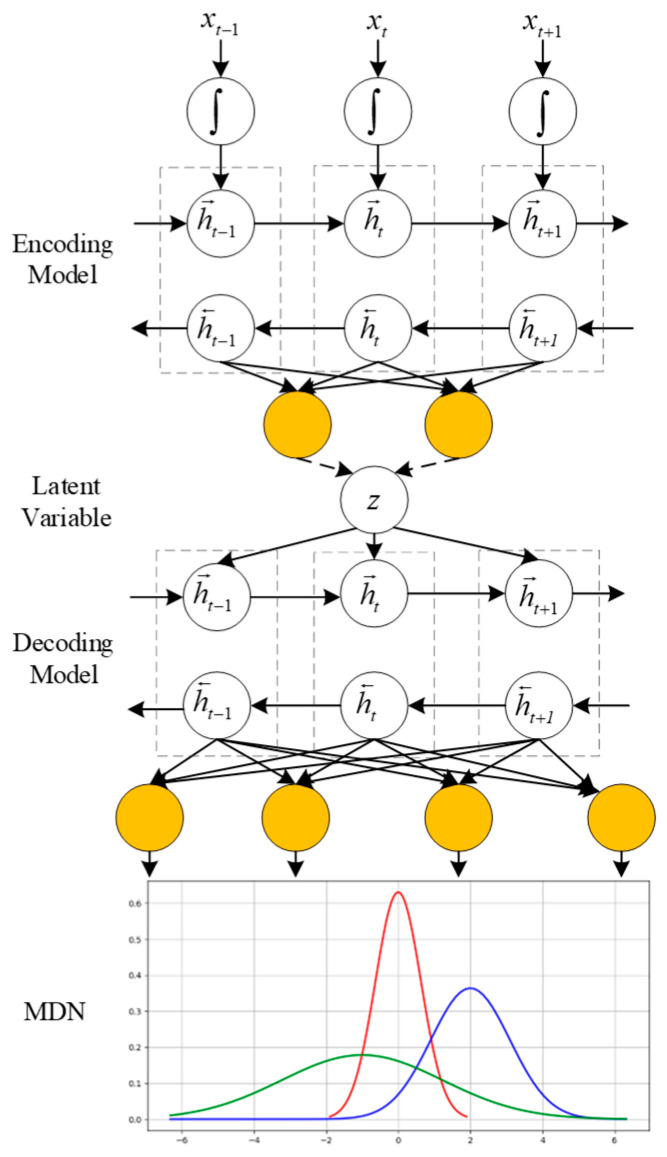
The architecture of the VAE-WRBM-MDN model. In the figure, the red curve represents our UAV’s dominance function value (corresponding to the advantageous situation *V*_1_), the blue curve denotes the target’s dominance function value (corresponding to the disadvantageous situation *V*_2_), and the green curve indicates the balanced confrontation state (corresponding to the mutual engagement situation *V*_3_).

**Figure 4:**
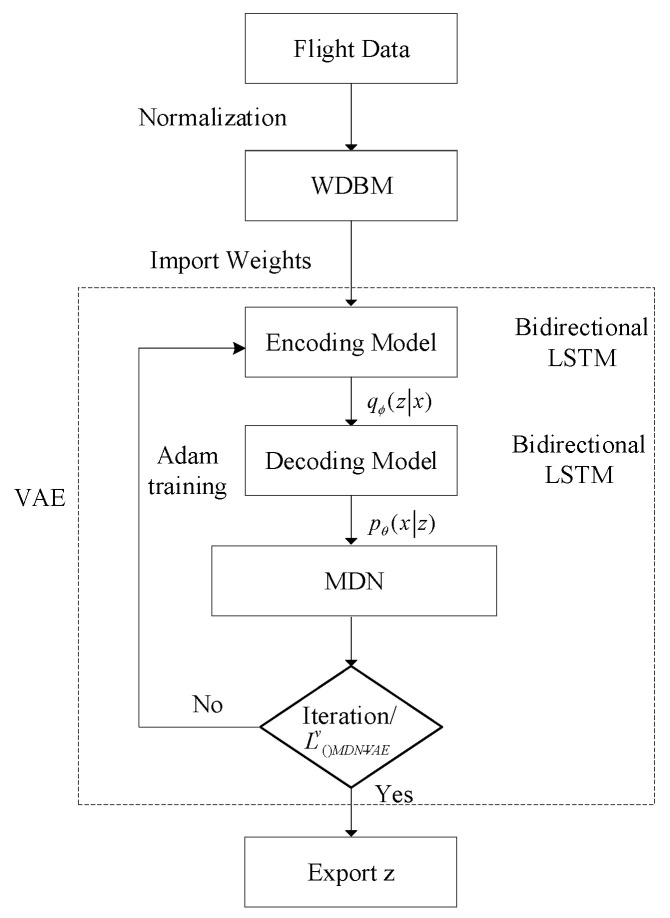
The training and inference workflow of the VAE-WRBM-MDN model.

The structure and flow chart of the improved VAE are shown in Figure 3 and Figure 4, respectively. As illustrated in Figure 4, the training process begins with layer-wise pre-training of the encoder and decoder weights using WRBMs (as detailed in Section 3.3). These pre-trained weights are then used to initialize the VAE-MDN model. The entire network is subsequently fine-tuned end-to-end using the Adam optimizer to maximize the objective function $L_{\left(MDN-VAE\right)}^{v}$ (Equation (7)). During inference, new flight data is passed through the trained encoder to obtain the latent representation z, which represents the extracted situational features.

## 4. Clustering with k-Means++ and Density Peak

After extracting low-dimensional latent features, we apply clustering algorithms to group them into distinct situational classes. We selected two representative algorithms: k-means++, as a robust and widely used baseline, and the density peak algorithm, which is adept at identifying clusters of arbitrary shapes and is less sensitive to initialization. The number of clusters was set to four, a choice directly motivated by the established air combat doctrine described in Section 2.2, which provides a tactically meaningful and interpretable partitioning of the situation space.

From Section 1, there are obvious differences in the feature values of four typical Relative state estimation situations, and extracted features corresponding to the same type of situation congregate together. Therefore, two typical clustering algorithms, including k-means++ and density peak are introduced to classify extracted situation features.

### 4.1. k-Means++

K-means++, proposed by Arthur and Vassilvitskii in 2007 [28], is an improved k-means algorithm in clustering centers. A new algorithm (Algorithm 1) is adopted in k-means++ to replace stochastic clustering centers. Compared with k-means, k-means++ achieves higher accuracy and speed.

**Algorithm 1** *k*-means++(*k*) initialization

$1\text{:}\;x^{\prime }\in X$


$\;\mathrm{sample}\;a\;\mathrm{point}\;\mathrm{uniformly}\;\mathrm{at}\;\mathrm{random}\;\mathrm{from}\;x^{\prime }\in X$

$2\text{:}\;\mathrm{while}\;x^{\prime }\in X$ do

$3\text{:}\;\mathrm{Sample}\;x^{\prime }\in X$


$\;\mathrm{with}\;\mathrm{probability}\;\frac{d^{2}(x^{\prime })}{\sum_{x\in X}d^{2}(x)}$



$4\text{:}\;C\leftarrow C\cup \left\{x^{\prime }\right\}$

5: end while

### 4.2. Density Peak

As for density peak [29], two vital features of data point i need to be achieved: local density $\rho_{i}$ and its minimum distance $\delta_{i}$ from points of higher density:

(8) $$\rho_{i}=\sum_{j}\chi (d_{ij}-d_{c})\;\chi (x)=\left\{\begin{array}{l} 1,x\leq 0\\ 0,x>0 \end{array}\right.$$

where $d_{c}$ represents cutoff distance, $\rho_{i}$ represents the number of points whose distance from point i is shorter than $d_{c}$, $\delta_{i}$ reflects the distance between points with higher density, which is defined as:

(9) $$\delta_{i}=\underset{j:\rho_{j}>\rho_{i}}{min}(d_{ij})$$

Center points of clustering should be surrounded by points with low density and keep a relatively longer distance from the other center points. Therefore, a data point can be considered as center point of clustering only if both $\rho_{i}$ and $\delta_{i}$ are over a certain threshold.

## 5. Construction of Knowledge-Based Heuristic Evaluation Functions for Data Refinement

Solely relying on data-driven clustering may lead to misinterpretations due to sensor noise or ambiguous states. To strictly align the classification with operational physics, we introduce a hybrid sensor fusion strategy. We refine the data-driven results using a knowledge-based Heuristic Evaluation Function derived from expert rules. This acts as a physics-guided constraint on the deep learning outputs. While calculating the Heuristic Evaluation Function values at some point, sample a total of 25 points forward and backward, whose weights are determined according to the interval from the moment. The closer the sample point is, the higher its weight is.

To ensure the reliability and objectivity of the expert knowledge used in this refinement step, a structured validation protocol was implemented. The “expert knowledge” was sourced from a panel of five subject matter experts (SMEs), each with over 10 years of experience in UAV operations and tactical analysis. The refinement process involved two key stages: First, the initial clustering results were independently reviewed by each SME against the calculated dominance function values and established tactical doctrines. Second, any discrepancies or ambiguous classifications were discussed by the panel in a consensus-building session to arrive at a final, validated classification. This structured, multi-expert approach, anchored by the quantitative dominance function, minimizes individual subjectivity and ensures the refined results are consistent with operational realities.

UAV’s System performance state and operational environment situation are two main aspects deliberated in situation awareness. Heuristic Evaluation Function $T_{c}$ of Relative state estimation capability is

(10) $$T_{c}=C/(\max(C))$$

(11) $$C=[\ln B+\ln(\sum A_{1}+1)+\ln(\sum A_{2})]\varepsilon_{1}\varepsilon_{2}\varepsilon_{3}\varepsilon_{4}$$

where C represents System performance state, B represents maneuvering parameters, $A_{1}$ represents onboard systems parameter, $A_{2}$ represents detection parameter, $\varepsilon_{1}$ represents steering coefficient, $\varepsilon_{2}$ represents Safety margin coefficient, $\varepsilon_{3}$ represents voyage coefficient and $\varepsilon_{4}$ represents Signal interference coefficient.

Angle Heuristic Evaluation Function $T_{a}$, distance Heuristic Evaluation Function $T_{d}$, speed Heuristic Evaluation Function $T_{v}$, and height Heuristic Evaluation Function $T_{h}$ are considered in Relative state estimation situation awareness. Weight coefficient is calculated by fuzzy analytic hierarchy process, synthesized situation Heuristic Evaluation Function T is obtained by weighted combination of five Heuristic Evaluation Functions:

(12) $$\left\{\begin{array}{c} T=k_{1}T_{c}+k_{2}T_{a}+k_{3}T_{d}+k_{4}T_{v}+k_{5}T_{h}\\ k_{1}+k_{2}+k_{3}+k_{4}+k_{5}=1 \end{array}\right.$$

where $k_{1}$, $k_{2}$, $k_{3}$, $k_{4}$, $k_{5}$ refer to weights of Relative state estimation capability advantage, angle advantage, distance advantage, speed advantage and height advantage, respectively.

**Figure 5:**
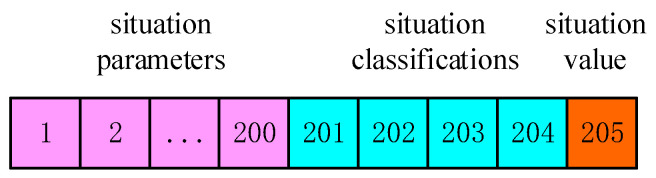
Format of data.

**Figure 6:**
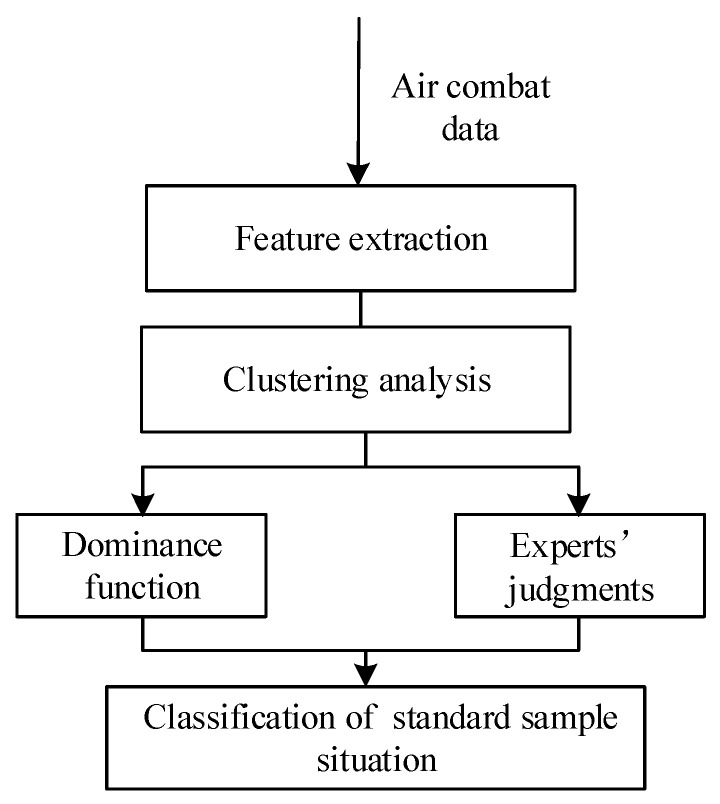
Flow chart of situation classification.

Storage form of specific situation awareness data is shown in Figure 5:

The specific process of situation classification is shown in Figure 6.

Figure 6 illustrates the complete workflow of our proposed situation classification framework. This hybrid approach is designed to ensure that the final results are both mathematically robust and operationally relevant. The process consists of two parallel streams that are ultimately synthesized:

1. Data-Driven Unsupervised Classification: The primary path begins with raw air combat data, which is fed into our deep feature extraction model (VAE-WRBM-MDN). The resulting low-dimensional features are then processed by a clustering algorithm (e.g., density peak) to generate an initial, purely data-driven classification of the situation.
2. Knowledge-Based Refinement: In parallel, the same data is used to calculate dominance function values, which provide a quantitative assessment based on established tactical principles. These values, combined with expert knowledge, serve as a reference for validating and correcting the initial clustering results.

The final and critical step involves fusing these two streams. The initial classifications from the clustering analysis are amended using the insights from the dominance function and expert judgments, particularly in ambiguous or rapidly changing situations where purely data-driven methods may falter. This synthesis ensures the final classification of the standard sample situation is accurate and aligned with real-world tactical realities.

## 6. Experiments

To validate the effectiveness and generalization capability of the proposed framework, all experiments were conducted on 10 distinct datasets. These datasets were generated from a high-fidelity aerial maneuvering simulator built upon the nonlinear six-degrees-of-freedom (6-DOF) equations of motion using Python 3.10. The simulation incorporates standard atmospheric models and aerodynamic coefficients to ensure the data reflects realistic physical flight dynamics Each dataset consists of 50,000 data points for training and 5000 for testing. This multi-dataset approach allows for a rigorous evaluation of the model’s performance across varied Sensor data patterns. The experimental hardware environment was a high-performance processor with 12 Intel Xeon(R) E5 CPU and 4GB RAM, and the software platform was Tensorflow 2.15.0.

### 6.1. Feature Extraction Experiments

We set a 4-layer VAE network by stacking BLSTMs to minimize information loss through hierarchical feature extraction. To determine the optimal network configuration and training parameters, we performed a systematic hyperparameter tuning process. We conducted a series of experiments by varying key parameters, including the network architecture (number of neurons in each layer), the optimizer’s learning rate, and the batch size, to identify the combination that yielded the best performance in terms of convergence speed and final loss value.

We specifically chose the Adam optimizer because it is an adaptive learning rate optimization algorithm that is well-suited for training deep neural networks with complex, non-stationary objectives like that of a VAE. Specifically, its adaptive nature is particularly effective for balancing the reconstruction loss and the KL divergence regularization term, which often have different gradient magnitudes.

It combines the advantages of both AdaGrad and RMSProp, providing efficient computation and requiring little memory, which leads to faster convergence in practice.

**Figure 7:**
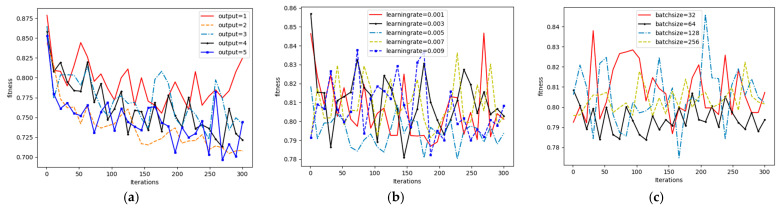
Hyperparameter tuning process for the VAE network. (**a**) Training loss with different network structures; (**b**) Training loss with different learning rates; (**c**) Training loss with different batch sizes.

The process involved a grid search over a predefined range of values. For instance, we evaluated several network structures (e.g., [1024, 256, 64, 2], [512, 128, 32, 2]), learning rates (e.g., 0.01, 0.005, 0.001), and batch sizes (e.g., 32, 64, 128). The training processes for these different parameter settings are illustrated in Figure 7. From these experiments, we concluded that a network structure of [1024, 256, 64, 2], combined with an Adam optimizer using a learning rate of 0.005 and a batch size of 64, achieved the most robust convergence and the lowest fitness value. This configuration was therefore selected for all subsequent experiments.

From Figure 7, it can be found that VAE-WRBM-MDN with the structure of [1024, 256, 64, 2] and an Adam optimizer with a learning rate of 0.005 and a batch size of 64 achieve robust and lower fitness.

According to the method above, optimal parameters of different networks can be reached: (1) WRBM: a learning rate of 0.001, a batchsize of 64, a dropout of 0.6 and iterations of 500. (2) VAE: a learning rate of 0.005, a batchsize of 64, a dropout of 0.6 and iterations of 300. (3) VAE-MDN: a learning rate of 0.005, a batchsize of 64, a dropout of 0.6 and iterations of 300.

**Figure 8:**
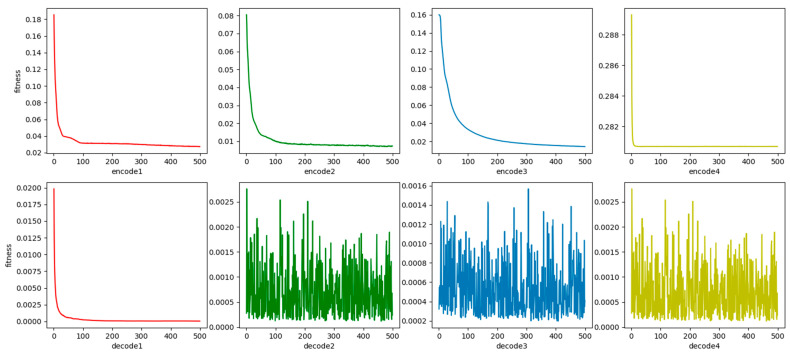
The training process of WRBM.

The red, green, blue, and yellow elements in Figure 8 correspond to the 1st to 4th layers of the WRBM: the curves in the upper subplots (marked with these colors) denote the training loss variation with iterations for the encoder layers (encode1 to encode4), while the columnar plots in the lower subplots (of the same colors) represent the loss fluctuations of the decoder layers (decode1 to decode4); this color-coding enables intuitive differentiation of convergence states across hierarchical WRBM layers, visually validating the effectiveness of layer-wise pre-training for providing stable initial weights to the subsequent VAE model. It can be seen in Figure 8 that WRBM is well-trained in respects of convergence speed and accuracy and effectively pretrains the initial weights of the VAE model.

**Figure 9:**
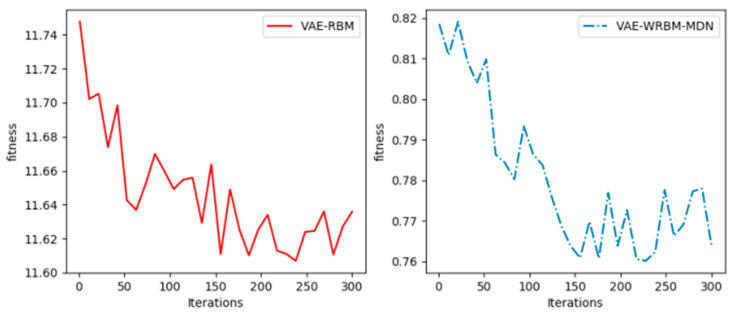
The training process of VAE-WRBM.

The training process of VAE-WRBM and VAE-WRBM-MDN is shown in Figure 9.

The training process of WRBM with the structure of [200, 1024], [1024, 256], [256, 64], [64, 2] and [2, 64], [64, 256], [256, 1024], [1024, 200] is shown in Figure 8:

From Figure 9, the loss of VAE-WRBM and VAE-WRBM-MDN converges rapidly to a relatively stable value, which demonstrates the efficiency of WRBM. However, in the experiment of VAE, we find its loss is always infinite. Through analysis, it is caused by stochastic, small initial weights and complex training data. Therefore, the optimization of weights by WRBM is indispensable to VAE models.

### 6.2. Classification Experiments

**Figure 10:**
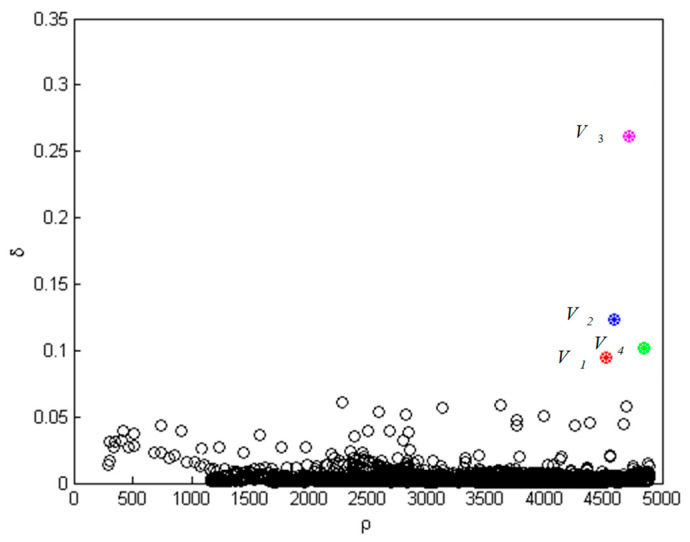
Value of density peaks algorithm.

**Figure 11:**
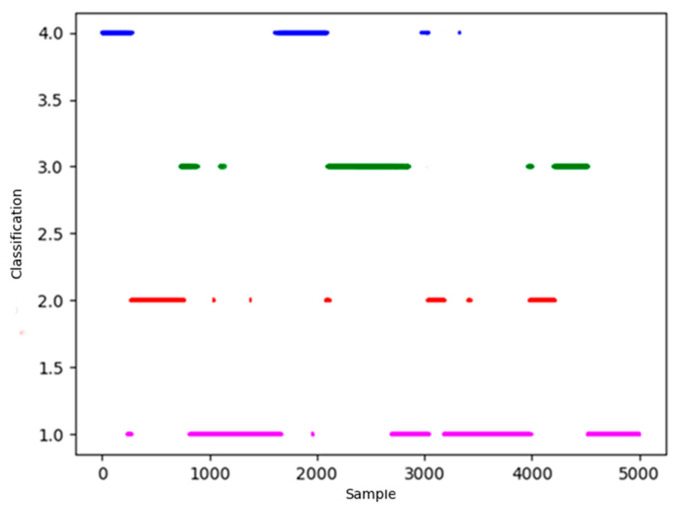
Cluster results of density peaks algorithm.

K-means++ and density peak methods are applied to classify the extracted features. In order to visualize the classification results, we take one set of test data as an example. The distribution results of density peak algorithm are shown in Figure 10:

In Figure 10, points of magenta, blue, green and red represent four centers of situation classification. Starting from four centers, take a certain distance as the classification radius to classify the remaining data. If there is an overlap, reclassify points in the light of the distance from the center points. The result of the classification is shown in Figure 11.

In Figure 11, blue, green and red represent four centers of situation classification. The horizontal axis represents 5000 test data involved in clustering, the vertical axis represents four types of situation. It is obvious that there are many discrete points in the classified results, which do not conform to the characteristic of situation continuity that need to be revised. 

According to Section 4, Heuristic Evaluation Function values of four types of data are calculated to determine the corresponding classification. Four colors are matched with four categories through Heuristic Evaluation Function and actual mission space situation: Magenta represents situation *V*_3_, red represents situation *V*_1_, green represents situation *V*_4_, and blue represents situation *V*_2_.

**Figure 12:**
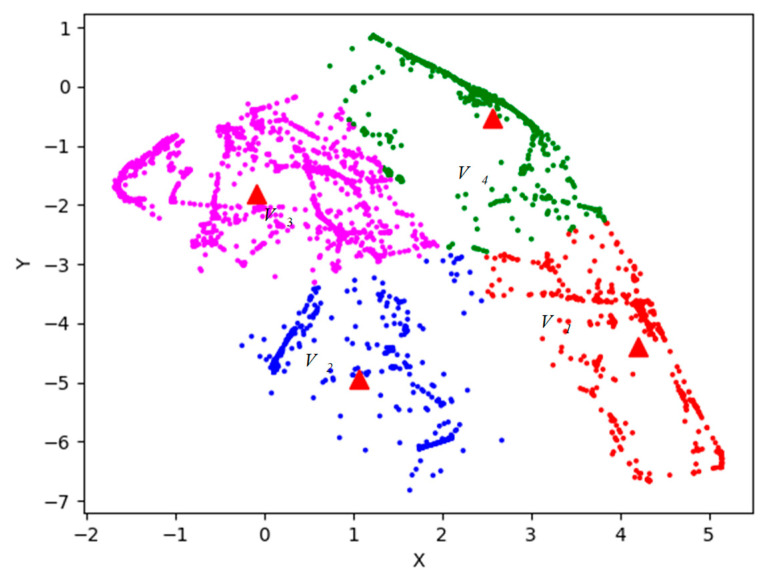
Value of k-means++ algorithm.

The distribution results of k-means++ algorithm are shown in Figure 12.

**Figure 13:**
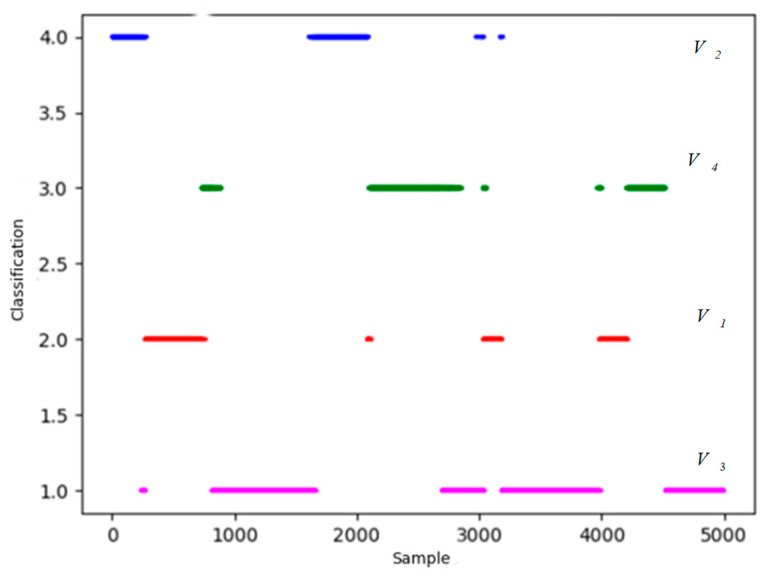
Cluster results of k-means++ algorithm.

In Figure 12, four red triangle points represent four clustering centers. Points of magenta, blue, green and red represent four types of situation. The specific classification result is shown in Figure 13.

**Figure 14:**
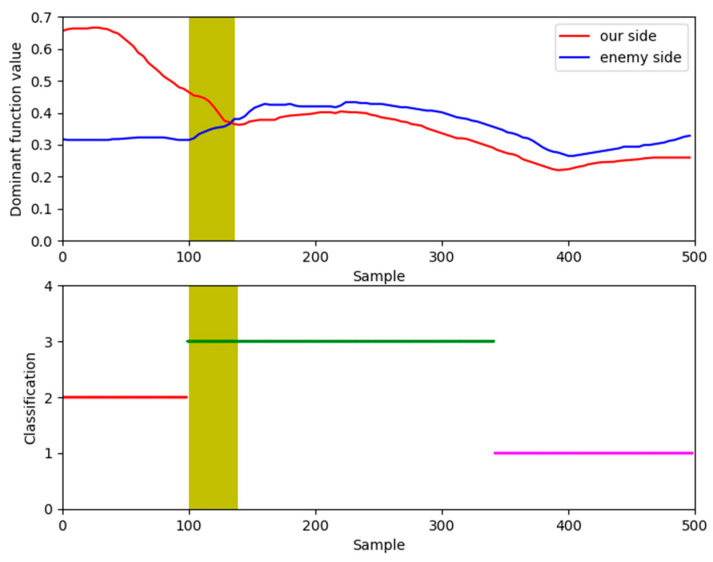
Comparison of two algorithms.

Similarly, in Figure 13 there are also some discrete points in the classified data, not in conformity with the physics of relative motion that need to be revised in light of the dominant values and experts’ perspectives. The Heuristic Evaluation Function values and clustering results of partial situation data are shown in Figure 14.

Figure 14 provides a compelling visual justification for the necessity of our refinement step by comparing the initial clustering results against the calculated dominance functions for a segment of the engagement. In the plot, the continuous red and blue lines represent the dominance function values for our UAV and the Target, respectively; a higher red line signifies our tactical advantage. The colored points at the bottom of the plot represent the situational class assigned by the unsupervised clustering algorithm.

**Figure 15:**
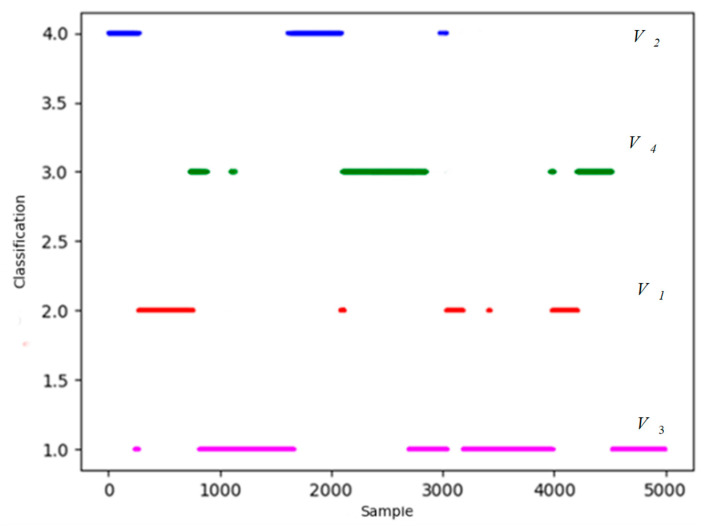
Final classification results of density peak algorithm.

**Figure 16:**
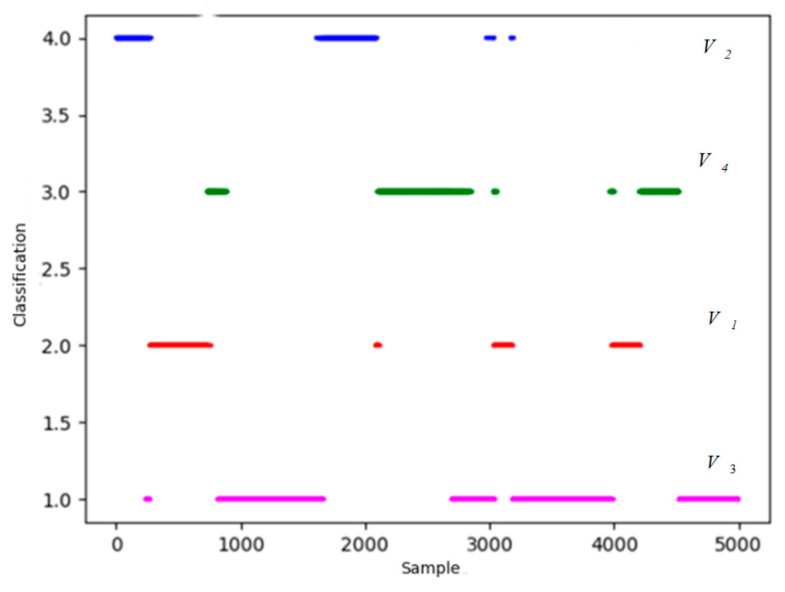
Final classification results of k-means++ algorithm.

The yellow shaded area highlights a critical discrepancy. Within this period, the dominance function clearly indicates that our UAV holds a significant advantage (the red line is consistently above the blue line). However, the clustering algorithm misclassifies this entire segment as a neutral situation (V3), failing to capture the shift in tactical advantage. Such errors are common in purely data-driven approaches, especially during transitional moments of an engagement. This discrepancy underscores the importance of the final refinement step, where the dominance function and expert knowledge are used to correct these misclassifications, as shown in the final results in Figure 15 and Figure 16, leading to a more accurate and operationally consistent situation assessment.

It is obvious that most discrete points are amended in Figure 15 and Figure 16. To verify the ability to extract features and classification accuracy, PCA is included to make the comparison. Follow the steps above, based on 10 sets of data, 10 comparative experiments are carried out. Training time and accuracy of the revised results are presented in Table 2.

As can be known from Table 2:

(1) The accuracy of the situation classification is low when k-means++ or density peak algorithm is directly used. It proves that the typical clustering algorithms are not suitable for high-dimensional data.
(2) As for test sets and training sets, compared with PCA, VAE-WRBM and VAE-WRBM-MDN achieve more favorable results, and VAE-WRBM-MDN outperforms VAE-WRBM. Since PCA is mainly designed for linear data, it is not adequate for complex nonlinear multi-dimensional data.
(3) Based on the extracted features, two typical clustering algorithms k-means++ and density peak both reach accurate classification results, and density peak performs better than k-means++. That implies that the extracted features can represent the whole dimension and the typical clustering methods are effective.
(4) Six algorithms all meet the requirements of timeliness and low hardware condition.

While Table 2 demonstrates the superior final classification accuracy of our method after knowledge-based refinement, it is also crucial to objectively evaluate the quality of the initial, purely data-driven clustering results. To address this, we introduced three standard external validity metrics: the Adjusted Rand Index (ARI), Normalized Mutual Information (NMI), and V-measure. We used the classification derived from the dominance function as the ground truth to assess how well the raw clusters (e.g., as visualized in Figure 13 and Figure 15) align with the desired situational categories.

The quantitative results of this evaluation are presented in Table 3. The data clearly shows that the features extracted by our VAE-WRBM-MDN model lead to significantly better initial clusters compared to the PCA baseline. For instance, our full model paired with density peak clustering achieved an ARI of 0.931 and an NMI of 0.945, far surpassing the values obtained by PCA. This provides strong, quantitative evidence that our model learns a latent representation where the underlying situations are inherently more separable. A high-quality initial clustering is fundamental, as it provides a more accurate foundation for the subsequent knowledge-based refinement, which in turn explains the high final accuracy reported in Table 2. This two-step validation confirms the effectiveness of our framework from both a data-driven and a final application perspective.

**Figure 17:**
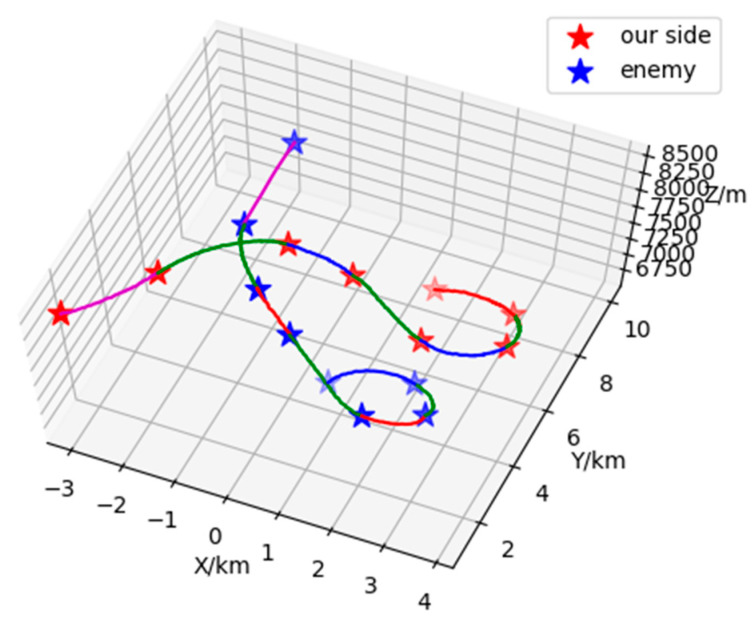
Three-dimensional situation of 1V1 in Relative state estimation.

**Figure 18:**
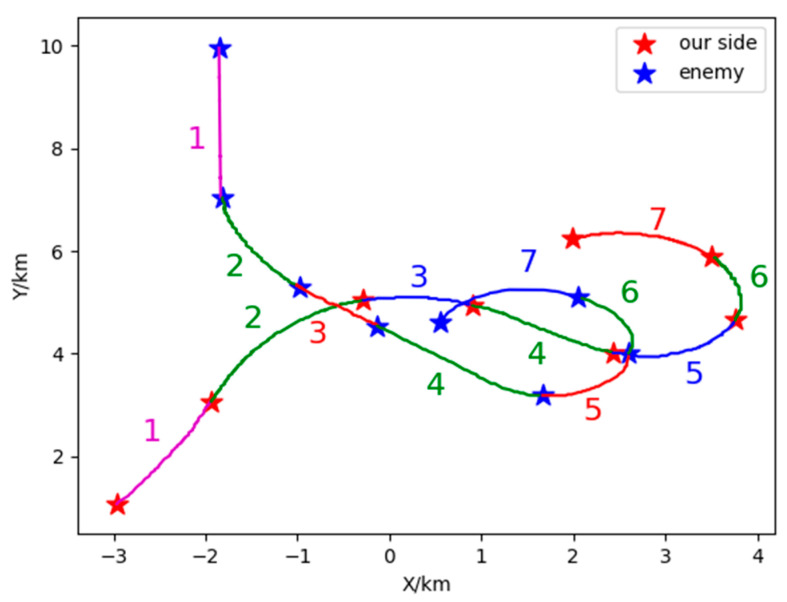
Two-dimensional of 1V1 in Relative state estimation.

Based on the combination of trained VAE-WRBM-MDN model and density peak algorithm, actual Relative state estimation data is processed and the Relative state estimation situation of each time period is classified. Furthermore, the classified results are compared with actual flight conditions to deeply analyze the Relative state estimation situation. Specific output results are shown in Figure 17 and Figure 18.

**Figure 19:**
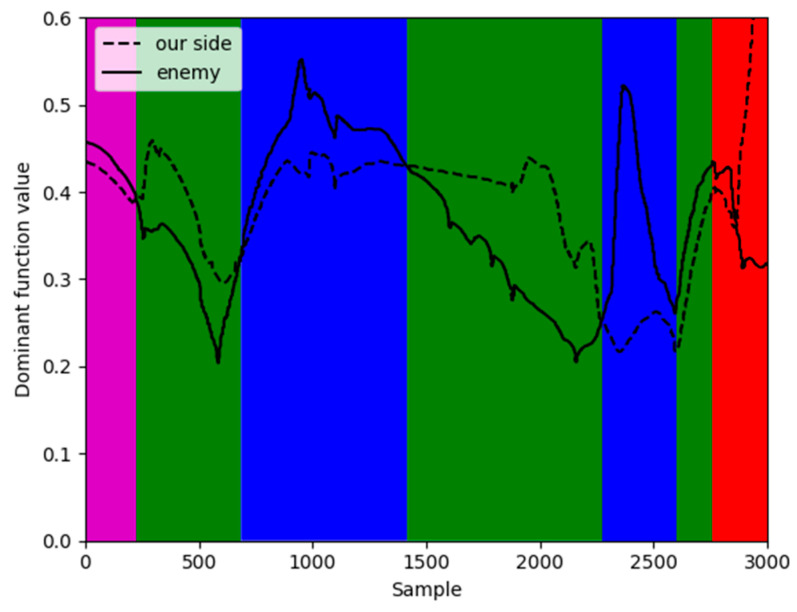
The situation value of Relative state estimation.

Figure 17 and Figure 18 show the classification results of two-dimensional and three-dimensional situation. Figure 19 displays the situation values and the corresponding classification results. In Figure 17 and Figure 18, red represents our UAV and blue represents the Target. The flight path is presented with red, blue, magenta and green in accordance with the situation *V*_1_~*V*_4_ respectively. The confrontation situation is divided into seven parts by the model. Part 1 corresponds to situation *V*_3_: at entry stage, the distance between both agents is large, far away from engage conditions; Part 2 corresponds to situation *V*_4_: both sides begin to maneuver and enter the confrontation area, threatening each other; Part 3 corresponds to situation *V*_2_: the Target emerges at the rear flank of our UAV, leaving us at a disadvantage. Part 4 corresponds to situation *V*_4_: our UAV has the advantage of altitude, while the Target occupy a slight advantage of angle, which contributes to the balance situation between both agents. Part 5 corresponds to situation *V*_2_: Target maneuvers heavily, expanding the advantage of the angle and narrowing the altitude gap. Therefore, our UAV is at a disadvantage. Part 6 corresponds to situation *V*_4_: both sides can maneuver to engage each other. Part 7 corresponds to situation *V*_1_: our UAV has the advantages of angle and altitude after Part 5 and Part 6, getting the opportunity to engage in the rear. Our UAV is at an advantage. The law of situation changes in Figure 19 is basically the same as the result of classification in Figure 17 and Figure 18. Therefore, the validity of the proposed model is verified in the actual confrontation process.

### 6.3. Ablation Study

To systematically validate our central hypothesis—that standard deep VAEs are highly unstable for this task and that our proposed components are essential for success—we conducted a rigorous ablation study. This study serves not only as an internal validation but also as the most critical state-of-the-art comparison, benchmarking our model against the foundational deep generative approach that any researcher would first attempt.

As noted in Section 6.1, the loss for the standard VAE model remained infinite, indicating a failure to converge due to suboptimal weight initialization. This result is from a new experiment conducted to isolate the effect of the BLSTM backbone.

The results clearly demonstrate the necessity of each component:

(1) Impact of WRBM: Overcoming a Fundamental Feasibility Barrier. The most dramatic and crucial finding of this study is the comparison between the standard VAE and our WRBM-enhanced version. The standard VAE, representing a direct and significant baseline, completely failed to converge, rendering it unusable for this task. The introduction of WRBM pre-training was not merely an incremental improvement; it was the enabling technology that made the entire deep generative modeling approach feasible, achieving a high accuracy of 94.86%. This confirms that our primary contribution is not just enhancing performance, but making it possible in the first place.
(2) Impact of BLSTM: By replacing the BLSTM backbone with a standard Multi-Layer Perceptron (MLP), the accuracy dropped significantly from 94.86% to 89.34%. This substantial decrease highlights the importance of capturing temporal dependencies in the UAV Time-series sensor data, which the BLSTM architecture is specifically designed to do.
(3) Impact of MDN: Adding the MDN to the VAE-WRBM model resulted in a further accuracy improvement from 94.86% to 95.69%. While the gain is more modest, it demonstrates that the MDN’s ability to model multi-modal output distributions provides a tangible benefit in representational power, leading to more precise feature extraction.

In summary, this ablation study validates our design choices, proving that the synergistic combination of WRBM for stability, BLSTM for temporal modeling, and MDN for expressive power is crucial for the high performance of our proposed framework.

## 7. Discussion

Our experimental results demonstrate that the proposed VAE-WRBM-MDN framework not only achieves high classification accuracy but also addresses fundamental challenges in applying deep generative models to real-world sensor data. This section provides a critical interpretation of these findings, discusses the study’s limitations, and outlines future research directions.

### 7.1. Interpretation and Implications of Key Findings

The most significant finding of this study is not merely the final accuracy figure, but the result from the ablation study (Table 4) showing that a standard VAE fails to converge. This is not a trivial point; it is a critical validation of our core hypothesis. It implies that for complex, non-linear time-series data typical of UAV engagements, applying deep generative models “out of the box” is not a viable strategy. The introduction of WRBM pre-training was not an incremental improvement but an enabling technology that transformed the VAE from an unusable theoretical construct into a practical and powerful feature extractor.

Furthermore, the performance gains from the BLSTM backbone and the MDN decoder highlight two other crucial aspects. The ~5% accuracy drop when replacing BLSTM with a simple MLP confirms that capturing temporal context is indispensable for situational understanding. The subsequent accuracy gain from adding the MDN, while more modest, demonstrates the benefit of modeling multi-modal distributions. This suggests that even within a single classified situation (e.g., “Mutual Engagement”), there exist multiple distinct sub-patterns in the sensor data, which our model can capture more effectively.

The broader implication of this work is a template for developing robust unsupervised learning systems for other complex autonomous platforms where labeled data is scarce or non-existent. The principle of stabilizing training (via WRBM), modeling temporal dynamics (via BLSTM), and capturing complex output distributions (via MDN) is a generalizable strategy.

### 7.2. Limitations and Future Work

Despite promising results, this study has several limitations that open avenues for future research.

Scenario and Environmental Complexity: The current framework was validated on a 1v1 engagement scenario under relatively clear conditions. Its performance in the presence of environmental factors like terrain masking, adverse weather, or electronic countermeasures remains untested. Future work must incorporate these real-world complexities into the simulation and validation datasets to enhance the framework’s operational robustness.

Scalability to Multi-Agent Systems: The model is designed for a single-target situation. Extending it to dynamic multi-UAV engagements, where the number of agents can vary, poses a significant challenge. The fixed-size input vector is insufficient for such scenarios. A promising direction is to integrate our VAE-WRBM-MDN backbone with Graph Neural Networks (GNNs). This would allow the model to represent the engagement as a dynamic graph, capturing the complex topological interactions between multiple agents and enabling scalable situation awareness in swarm scenarios.

Benchmarking Against Other Unsupervised Methods: While our framework significantly outperforms PCA and a baseline VAE, a broader comparison against other state-of-the-art unsupervised methods would further strengthen our findings. Future work should benchmark our model against advanced deep clustering algorithms (e.g., Deep Embedded Clustering, DEC) and contrastive learning approaches (e.g., SimCLR adapted for time-series data). This would provide deeper insights into the relative merits of generative versus discriminative paradigms for this specific task.

Transition to Real-World Deployment: Deploying this model onto a physical UAV requires addressing the significant constraints of onboard computation and real-time processing. The path to deployment involves model optimization through techniques like quantization and pruning, followed by implementation on specialized embedded hardware (e.g., NVIDIA Jetson, FPGA). Rigorous Hardware-in-the-Loop (HIL) simulation will be a critical intermediate step to validate the real-time performance and safety of the deployed system before any flight tests.

## 8. Conclusions

This paper introduced a novel, fully unsupervised situation awareness framework to overcome the critical challenges of applying deep generative models to high-dimensional UAV sensor data. By synergistically combining a Bidirectional Long Short-Term Memory (BLSTM) backbone, a Weighted-uncertainty Restricted Boltzmann Machine (WRBM) for pre-training, and a Mixture Density Network (MDN) in the decoder, our proposed VAE-WRBM-MDN model achieves both stable training and superior representational power.

Our key contributions are threefold: (1) We demonstrated that WRBM pre-training is an essential step to overcoming the training instability that renders standard VAEs unusable for this task. (2) We enhanced the model’s expressiveness by integrating an MDN, enabling it to capture the complex, multi-modal nature of engagement data. (3) We developed a hybrid classification process that refines the unsupervised clustering results with knowledge-based functions, ensuring the final assessment is both data-driven and operationally sound.

Extensive experiments confirmed the superiority of our approach, achieving a classification accuracy of 95.69%. The results validate that our framework provides a robust and generic solution for intelligent sensor data interpretation, paving the way for more autonomous and reliable UAV operations in complex environments.

## Figures and Tables

**Table 1:** Nomenclature for Situational Parameters.

Signal	Name	Range
λ	azimuth angle	$\left[0,\pi \right]$
ψ	entry angle	$\left[0,\pi \right]$
η	angle between velocity vectors	$\left[0,\pi \right]$
r	distance	$\left[0,10\right]\mathrm{km}$
$\Delta v^{2}$	variance of relative velocity	—
v	speed of our UAV	—
Δh	altitude difference	$\left[0,18,000\right]\;m$
h	altitude of own UAV	$\left[0,18,000\right]\;m$

**Table 2:** Comparison of classification.

Classified Method	Accuracy Rate (Train/Criterion)/%		Total Run Time/min	Run Time of a Single Data/s	Peak of CPU Occupancy Rate/%
	Train	Test			
k-means++	75.42	73.39	8	0.07	0.26
PCA+k-means++	81.79	80.37	9	0.10	0.31
VAE+k-means++	0	0	Non	Non	Non
VAE-WRBM+k-means++	95.21	94.50	372	0.14	0.83
VAE-WRBM-MDN+k-means++	95.98	95.43	401	0.18	1.07
density peak	76.13	75.21	20	0.09	0.61
PCA+density peak	82.41	81.53	22	0.13	0.87
VAE+density peak	0	0	Non	Non	Non
VAE-WRBM+density peak	95.41	94.86	380	0.37	0.94
VAE-WRBM-MDN+density peak	96.07	95.69	410	0.45	0.99

**Table 3:** Quantitative evaluation of initial clustering performance using external validity metrics.

Feature Extractor	Clustering Algorithm	Adjusted Rand Index (ARI) ↑	Normalized Mutual Information (NMI) ↑	V-Measure ↑
PCA	k-means++	0.654	0.681	0.680
PCA	density peak	0.672	0.695	0.694
VAE-WRBM	k-means++	0.885	0.893	0.893
VAE-WRBM	density peak	0.891	0.902	0.902
VAE-WRBM-MDN	k-means++	0.923	0.930	0.930
VAE-WRBM-MDN	density peak	0.931	0.945	0.945

**Table 4:** Ablation study results comparing different model configurations.

Model Configuration VAE (BLSTM Backbone)	Description Baseline VAE Without WRBM or MDN	Test Accuracy (%) Failed to Converg	Key Finding A Standard Deep VAE Is Unstable for This Task
VAE (BLSTM backbone)	Baseline VAE without WRBM or MDN	Failed to Converg	A standard deep VAE is unstable for this task
VAE-WRBM (BLSTM backbone)	VAE with WRBM pre-training	94.86	WRBM is essential for stable training and convergence
VAE-WRBM (MLP backbone)	VAE-WRBM with Feed-Forward layers	89.34	BLSTM effectively captures temporal features, significantly boosting accuracy
VAE-WRBM-MDN (BLSTM backbone)	Our full proposed model	95.69	MDN further improves accuracy by modeling complex data distributions

## Data Availability

The data presented in this study are available on request from the corresponding author due to privacy concerns.

## DURC Statement

Current research is limited to the UAV Sensor Data Fusion, which is beneficial and does not pose a threat to public health or national security. Authors acknowledge the dual-use potential of the research involving UAV Sensor Data Fusion Collaboration and confirm that all necessary precautions have been taken to prevent potential misuse. As an ethical responsibility, authors strictly adhere to relevant national and international laws about DURC. Authors advocate for responsible deployment, ethical considerations, regulatory compliance, and transparent reporting to mitigate misuse risks and foster beneficial outcomes.
